# Trisomy Xp and partial tetrasomy Xq resulting from gain of a rearranged X chromosome in a female fetus: pathogenic or not?

**DOI:** 10.1186/s13039-015-0160-5

**Published:** 2015-07-25

**Authors:** Maria Yiu, Zhongxia Qi, Anita Ki, Jingwei Yu

**Affiliations:** Department of Laboratory Medicine, University of California, San Francisco. 185 Berry Street, Suite# 290, Campus Box 0134, San Francisco, CA 94107 USA; Clinical Cytogenetics Laboratory, UCSF Medical Center, San Francisco, CA USA

**Keywords:** Prenatal diagnosis, Sex chromosome abnormality, Array CGH, X inactivation, High risk pregnancy, Genetic counseling, Chorionic villus sampling, Amniocentesis

## Abstract

Cytogenetic analysis of chorionic villous sampling revealed a mosaic karyotype with gain of a rearranged X chromosome. Microarray and additional studies indicated that the rearranged X carried an inverted duplication, a deletion and a satellited Xqter. Gain of this rearranged X was confirmed by follow-up amniocentesis and postnatal cord blood sample. A full-term infant girl was delivered and showed normal physical findings at both birth and 21-month follow-up examinations. Late replication studies demonstrated that the rearranged X was inactivated in all abnormal cells analyzed. Skewed X-inactivation may suppress the potentially deleterious effects of genomic imbalance; however, gain of X chromosomes, particularly rearranged X chromosomes, often presents challenges for prenatal genetic counseling. The gradation of clinical phenotype severity generally correlates with the number of additional X chromosomes. However, the X chromosome regions responsible for the abnormal phenotypes are poorly understood. This case will further elucidate the phenotypic effects of X inactivation and X chromosome abnormalities.

## Background

Sex chromosome aneuploidies are considered the most common chromosomal abnormalities compatible with live births. A recent report suggested that the prevalence of sex chromosome trisomies is 0.19–5.36 per 1000 in European countries [1].

Sex chromosome abnormalities generally have less deleterious clinical effects as compare to autosomal aberrations. However, selective inactivation of the structurally rearranged X chromosome does not inevitably confer phenotypic normalcy on females. The presence of a complex rearranged third X chromosome often raises concern for the phenotypic significance of genomic dosage alteration and skewed X-inactivation. The wide phenotypic variation and lack of long term follow-up reports in current literature make definitive prognosis for genetic counseling more challenging and problematic. We report a normal female infant after the prenatal diagnosis of an extra satellited X chromosome with trisomy Xp and partial tetrasomy Xq.

## Case presentation

A primiparous woman elected for chorionic villous sampling (CVS) with direct Fluorescence in situ hybridization (FISH) testing due to advanced maternal age. Family history was noncontributory. Cytogenetic analysis revealed a mosaic karyotype with gain of a rearranged X; *47,XX,add(X)(q?22)[11]/46,XX[9]*. Both parental karyotypes were normal. The rearranged X chromosome was further characterized by array CGH, FISH, Ag-NOR, and X-late replication analysis. Following the diagnosis of an abnormal female karyotype, the pregnancy was monitored by serial ultrasound examinations and fetal echocardiograms, which revealed a normal female fetus. A healthy baby girl was delivered at 39 ^4/7^ week’s gestation. Genetics evaluations at birth and 21 months of age determined that the baby presented with age-appropriate growth and development.

## Results

### Chromosome analysis

**Fig. 1:**
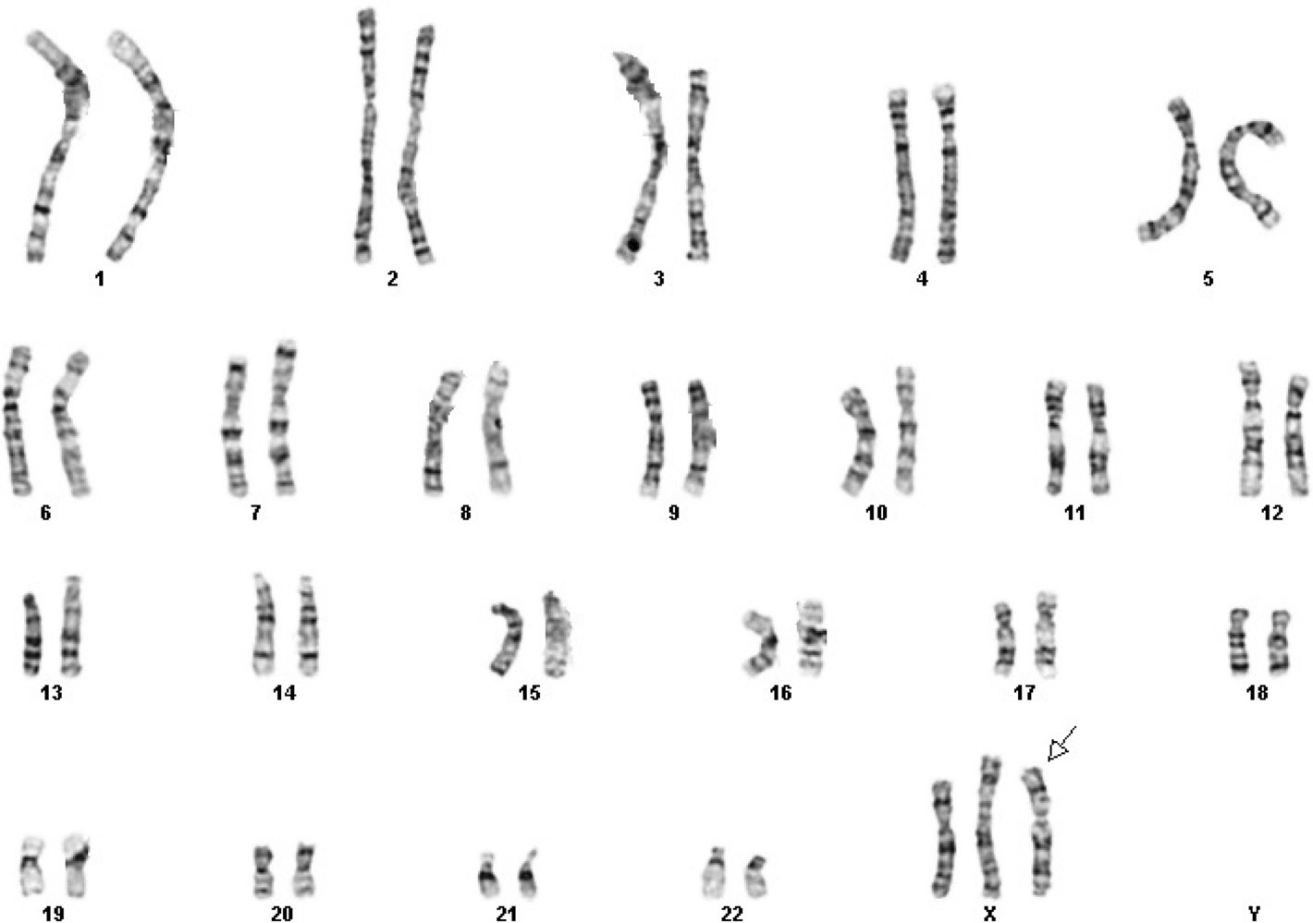
GTG banding showed gain of an abnormal X chromosome with satellites. Analysis of chorionic villus, amniotic flid and cord blood cells revealed an abnormal X (arrow)

The chorionic villus cell karyotype was *47,XX,add(X)(q?22)[11]/46,XX[9]* from four different initial cultures. Both of parental chromosomal studies were normal. Follow-up amniocentesis indicated full aneuploidy with an additional der(X) chromosome in all available 54 colonies; *47,XX,der(X)del(X)(q22.1)dup(X)(q22.1q11.1)* (Fig. 1). Prenatal ultrasonography imaging including fetal echocardiography was unremarkable. Subsequent high resolution G-band study of postnatal cord blood showed 92 % of the analyzed cells with an extra satellited chromosome; *47,XX,der(X)del(X)(q22.1) dup(X)(q22.1q11.1) t(X;?)(q11.1;p11.2).*

### Cytogenomic microarray

**Fig. 2:**
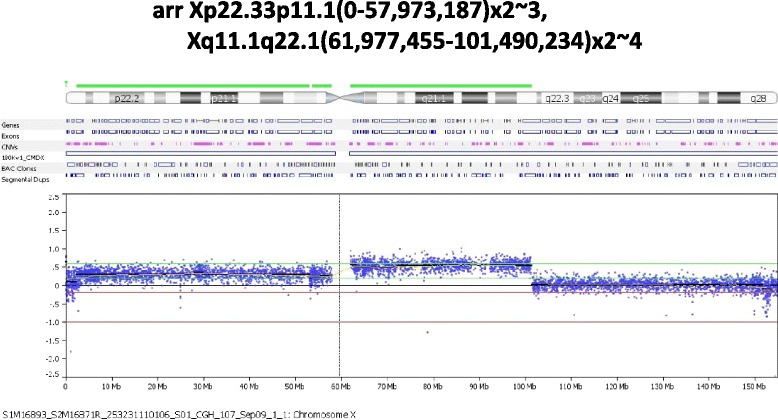
Chromosome microarray analyses on chorionic villus cells. The results demonstrated a single copy gain of 55 Mb on the Xp arm and two copies gain of 39.5 Mb of Xp11.1-q22.1. The Xq22.1-qter did not show a gain of material

Oligonucleotide array CGH of chorionic villus cells showed *arr Xp22.33p11.1(2,327,474–57,973,187)x2 ~ 3, Xq11.1q22.1(61,977,455–101,490,234)x3* (Fig. 2), confirming mosaicism of a single copy gain on the Xp arm and possible 2 copies gain of the Xp11.1-q22.1.

### Fluorescence in situ hybridization study

**Fig. 3:**
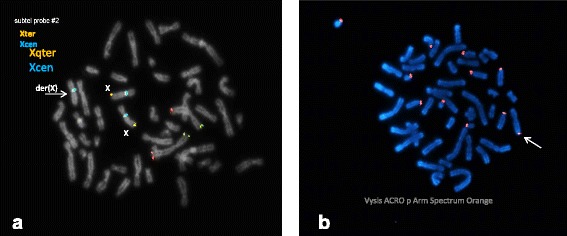
FISH analysis with subtelomere (*yellow*) and satellite DNA (*red*) probes. **a**: Two normal X chromosomes showed positive staining and der(X) showed negative staining. **b**: Der(X) chromosome showed an extra signal

Direct FISH analysis of CVS cells revealed a female XXX signal pattern in 37 of 50 interphase nuclei scored; the remaining 13 nuclei showed a normal XX signal pattern. Subtelomere FISH revealed a negative hybridization of the long arm of the der(X) chromosome, indicating deletion of the Xqter region (Fig. 3a). A few cells with higher resolution analysis revealed an appearance of satellites located on the telomeric long arm of der(X). Ag-NOR stain was positive for acrocentric nucleolus organizer regions (Fig. 3b). Both of parental chromosomal studies were normal and showed no evidence of a satellited Xq.

### X-inactivation assay

**Fig. 4:**
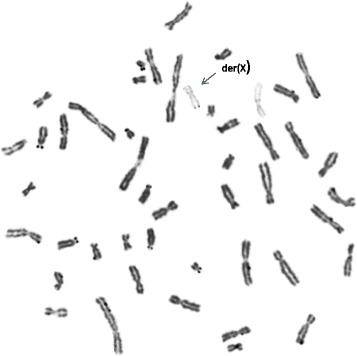
Late replication of the rearranged der(X) and a normal X chromosome by BrdU replication analysis and Ag-NOR staining

X chromosome late replication pattern of the villi cells using terminal 5- bromodeoxyuridine pulse [2] revealed delayed replication of der(X) and one of the normal X chromosomes (Fig. 4).

### Short tandem repeat analysis

**Fig. 5:**
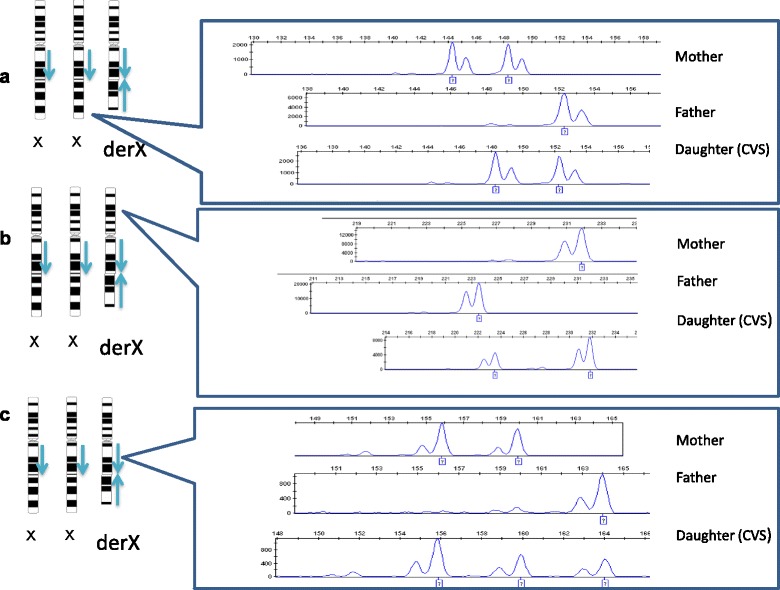
**a**,**b**,**c**: Short tandem repeat analysis. **a**: Marker DXS7133 showed biparental inheritance of the two normal X chromosomes and **b**: Marker DXS6807 on Xp demonstrated a 2:1 peak ratio corresponding to one maternal allele and one paternal allele and **c**: Marker DXS6789 (within the duplication region) indicated 2 identical maternal alleles with a 2:1 intensity ratio and one paternal allele

Polymorphic short tandem repeat markers, DXS6807, DXS6789, and DXS7133,were selected from ChrX-STR.org2.0 (www.chrx-str.org) to correspond with the genome region in the short arm of X chromosome, and the duplicated and deleted long arm of the der(X) chromosome, respectively. Results of the STR analysis on fetal and parental DNA are summarized as follows: marker DXS7133 on del(Xq) terminus showed one paternal and one maternal allele and indicated biparental inheritance of the normal two X chromosomes (Fig. 5a); marker DXS6807 on Xp demonstrated a 2:1 ratio of maternal allele versus paternal allele (Fig. 5b), indicating the der(X) is of maternal origin; marker DXS6789 detected three distinct alleles within the duplicated X region indicating two maternal X chromosomes of meiotic origin were involved in the rearrangement (Fig. 5c).

## Discussion

The majority of cytogenetically detectable unbalanced rearrangements are associated with significant phenotypic abnormalities. Unbalanced rearrangements with additional sex chromosomes often present challenges for informed genetic counseling. We present a prenatally diagnosed supernumerary X chromosome with complex rearrangements of inverted duplication with deletion and gain of satellites in a phenotypically normal female infant.

**Fig. 6:**
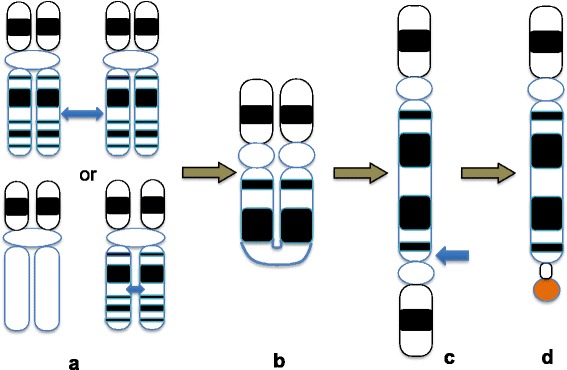
Proposed mechanism for the origin of the extra rearranged X chromosome. **a**: Interchromatid exchange in maternal meiosis, **b**: formation of a dicentric chromosome, loss of the acentric fragments, **c**: separation of two centromeres at anaphase resulting in breakage near one of the centromeres, **d**: inv dup(X) with del(X) and satellited Xq. Blue arrows indicate breakpoints

Several mechanisms have been reported for the formation of inverted duplication with terminal deletion [3–7]. In this present case, an illegitimate crossing-over event took place between two maternal X homologous chromosomes at Xq22.1. The arising dicentric recombination was a segment of Xpter ->cen ->Xq22.1::Xq22.1 ->cen ->Xpter. The acentric Xq22.1 ->qter segment was lost in the process. At subsequent cell division, a breakage between two centromeres occurred at Xq11.1, resulting in an inverted duplication and an uncapped terminal deletion. A healing of the broken end with acrocentric satellite DNA would presumably follow (Fig. 6).

A satellited chromosome is a rare chromosomal anomaly and most reported de novo cases are associated with complex structural rearrangements [8, 9]. Scattered within the genome are repeat sequences with homology to the acrocentric repeat DNA. Homology between the repetitive sequences and spatial proximity in a common nucleolus may favor recombination. Various DNA end repair mechanisms may also play an important role in stabilizing terminal deletions with repetitive DNA-sequence elements [10–14].

In contrast to trisomy X with three structurally normal X chromosomes and random inactivation, the present karyotype has two copies of Xq22-qter, three copies of Xp, four copies of Xq11.1-q22 and skewed X inactivation. Genotype-phenotype correlations in individuals with extra copies of Xq and Xp are variable [15–26]. The presence of a normal phenotype and the identification of non-random X-inactivation of the abnormal X suggest that the inactivation minimizes the deleterious effect of structural and genomic imbalance. However, the presence of normal cells in other tissues is unknown.

There is no literature on the reproductive experience of individuals with the present chromosomal constitution. This karyotype might be associated with an increased risk of offspring with sex chromosome abnormality. Correlation with other clinical findings and characterization of the structural rearrangement at molecular level will assist in understanding the precise impact of rearranged supernumerary X and its role in phenotypic effects.

## Conclusions

The clinical significance of gain of an X chromosome with a complex structural rearrangement containing four copies of the proximal Xq11.1-Xq22.1 region has not been previously described. Characterization of the structural rearrangement at molecular level further elucidated the phenotypic effects of X inactivation and X chromosome abnormalities. The identification of non-random X-inactivation of the abnormal X chromosome suggests that the inactivation minimizes the negative effects of the structural and genomic imbalances. The findings from this study demonstrated the importance of additional testing in the cases with unbalanced X chromosome rearrangements in prenatal genetic counseling.

## Consent

Written informed consent for this case report was obtained from the patient.

A copy of the written consent is available for review by the Editor-in-Chief of this journal.
